# Optical Fiber Magnetic Field Sensors Based on 3 × 3 Coupler and Iron-Based Amorphous Nanocrystalline Ribbons

**DOI:** 10.3390/s23052530

**Published:** 2023-02-24

**Authors:** Minggan Lou, Wentao Zhang, Wenzhu Huang, Xuekui Xi

**Affiliations:** 1 State Key Laboratory of Transducer Technology, Institute of Semiconductors, Chinese Academy of Sciences, Beijing 100083, China; 2 Center of Materials Science and Optoelectronic Engineering, University of Chinese Academy of Sciences, Beijing 100049, China; 3 Shenzhen Academy of Disaster Prevention and Reduction, Shenzhen 518003, China; 4 Beijing National Laboratory for Condensed Matter Physics, Institute of Physics, Chinese Academy of Sciences, Beijing 100190, China

**Keywords:** optical fiber, magnetic field sensor, 3 × 3 coupler, magnetostrictive effect, equal-arm interferometer

## Abstract

Optical fiber interferometric magnetic field sensors based on magnetostrictive effects have several advantages, e.g., high sensitivity, strong adaptability to harsh environments, long distance transmission, etc. They also have great application prospects in deep wells, oceans, and other extreme environments. In this paper, two optical fiber magnetic field sensors based on iron-based amorphous nanocrystalline ribbons and a passive 3 × 3 coupler demodulation system were proposed and experimentally tested. The sensor structure and the equal-arm Mach–Zehnder fiber interferometer were designed, and the experimental results showed that the magnetic field resolutions of the optical fiber magnetic field sensors with sensing length of 0.25 m and 1 m were 15.4 nT/√Hz @ 10 Hz and 4.2 nT/√Hz @ 10 Hz, respectively. This confirmed the sensitivity multiplication relationship between the two sensors and the feasibility of improving the magnetic field resolution to the pT level by increasing the sensing length.

## 1. Introduction

Magnetic field detection plays an essential role in national defense security, agriculture, resource exploration, geophysics, and other fields [1]: for example, submarine detection in the military, stratum analysis in geophysics, the exploration of iron and nickel resources in industry, earthquake monitoring, navigation, magnetic plasmon modes for efficient refractive index sensing, etc. [2,3,4]. The magnetic field sensors include fluxgate magnetometer, optically pumped magnetometers, Hall-effect sensors, superconducting quantum interference device magnetometer, Magnetoelectric sensors, etc. [5,6]. The magnetoelectric sensors exhibit a simple structure, low cost, and high sensitivity. Moreover, magnetoelectric sensors have been one of the important development directions in recent years [6]. In many advanced fields, such as magnetic anomaly detection, optical frequency domain reflection [3], magnetic imaging [7,8], human magnetic field detection [9], etc., magnetic field sensors are required to achieve magnetic field resolutions of the nT or even pT level in the low frequency band (hundreds of Hertz to hundreds of seconds), and to improve system detection capabilities, image resolution, etc. For example, the stray magnetic fields emitted from the human being are of the order of 1–100 pT from the DC to 100 Hz [6]. Traditional magnetic field sensors have been able to detect a minimum magnetic field of 10^−14^ T at 1 Hz [10]. However, traditional magnetic field sensors require current and their applications in some extreme environments, such as wells or seabeds, are also limited. Optical fiber magnetic field sensors have attracted extensive research attention due to their advantages of high sensitivity, small size, strong adaptability to harsh environments, long distance transmission, etc., over the past few decades [11,12,13,14]. 

There are four main types of optical fiber magnetic field sensors: they are based on the Faraday effect, magnetic fluids, the magnetostrictive effect, and Lorentz force, respectively [15,16,17,18]. In 1980, the possibility of optical fiber magnetic field sensors based on the magnetostrictive effect (OFMSME) was first proposed by Yariv and Winsor, who showed that the minimum detectable magnetic field was 1.6 × 10^−12^ G theoretically [19]. An OFMSME with passive demodulation and high theoretical magnetic field resolution would have significant application prospects in complex environments such as deep wells and oceans. For OFMSMEs, the main problems are that magnetic saturation, hysteresis, and other properties of magnetostrictive materials—along with environmental noise and thermal noise—affect system stability and limit resolution. The magnetic field resolution of the OFMSME was expressed as:

(1) $$H_{\min}=\varphi_{\min}/K,$$

where *H*_min_ is the magnetic field resolution of the OFMSME, *φ*_min_ is the phase resolution, and *K* is the sensitivity. Therefore, many research studies have been carried out, focused on improving sensitivity and demodulation resolution and improving the magnetic field resolution [11,20,21,22,23,24,25]. Y.W.Bibby et al. achieved a sensitivity of 0.0091 rad/μT by applying a bias magnetic field and using an optical fiber coated with a magnetostrictive film [21]. Feifei Chen and Yi Jiang et al. used a TbDyFe rod to design a magnetostrictive sensitization winding structure, which achieved a sensitivity of 0.2581 rad/μT and a phase resolution of 9.17 × 10^−5^ rad/√Hz @ 200 Hz by winding 30 m fiber [22]. Phase-locked technologies have often been used to suppress system noise [11,23,24,25]. Nevertheless, those active demodulation methods require current at the sensing part, which limits the bandwidth and weakens the advantages of a passive demodulation system. A passive demodulation system also requires further reductions in noise level in order to improve the magnetic field resolution at low frequencies. 

In this paper, two optical fiber magnetic field sensors based on iron-based amorphous nanocrystalline ribbons and a passive 3 × 3 coupler demodulation system were proposed and experimentally tested. The stiffness coupling model between the iron-based amorphous nanocrystalline ribbons and fiber was established, and the sensor structure and equal-arm Mach–Zehnder fiber interferometer were designed. Then, the linearity, frequency response, and directivity of the sensors were tested. The experimental results showed that the magnetic field resolutions of the optical fiber magnetic field sensors with sensing length of 0.25 m and 1 m were 15.4 nT/√Hz @ 10 Hz and 4.2 nT/√Hz @ 10 Hz, respectively. This confirmed the sensitivity multiplication relationship between the two sensors and the feasibility of improving the magnetic field resolution by increasing the sensing length.

## 2. Methods

### 2.1. System Configuration

**Figure 1:**
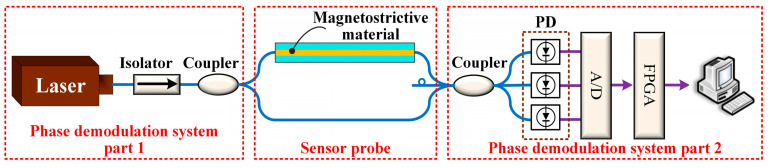
The optical fiber magnetic field sensors based on the magnetostrictive effect.

The optical fiber magnetic field sensing system is shown in Figure 1. The system consisted of two parts: the sensor probe, based on iron-based amorphous nanocrystalline ribbons, and the passive 3 × 3 coupler demodulation system.

The sensor probe was a Mach–Zehnder fiber interferometer, composed of a 1 × 2 coupler and a 3 × 3 coupler. Both the 1 × 2 coupler and the 3 × 3 coupler adopted bending-resistant fibers. The lengths of the reference arm and the sensing arm were equal (see Appendix A), which effectively reduced the frequency noise of the light source, thermal noise, and ambient temperature influence. The magnetic field sensitive material was iron-based amorphous nanocrystalline (Fe_78_Si_13_B_9_), which demonstrated high sensitivity to the magnetic field. The Fe_78_Si_13_B_9_ material was made into a thin ribbon, and the fiber could be pasted or wound repeatedly along the ribbon to achieve a longer sensing length.

The all-optical system used passive demodulation based on a 3 × 3 coupler and had no additional carrier signal. The light was emitted from the laser and split via a 1 × 2 coupler. Only the light entering the sensing arm was modulated by the Fe_78_Si_13_B_9_. Then, the two beams of light were set to interfere in the 3 × 3 coupler. Finally, three signals with a phase difference of 120° were output. The three signals were converted into electrical signals by photodetectors, and the voltage signals were collected by the 9223 module of the cRIO system. Then, the collected signals were demodulated in LabVIEW, and finally transmitted to the computer for display.

### 2.2. Theoretical Derivation

**Figure 2:**
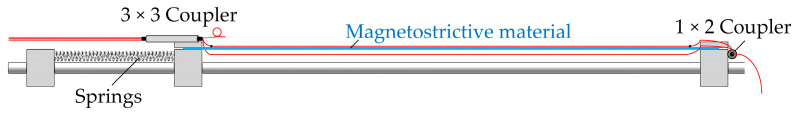
The fiber magnetic sensor structure design.

The sensor structure was designed as shown in Figure 2. Only the middle stainless-steel block was movable, and the Fe_78_Si_13_B_9_ ribbons were stretched by springs and stainless-steel blocks. Using a spring to stretch the magnetostrictive material served to keep the Fe_78_Si_13_B_9_ ribbons in a state of tension, so that the strain of the Fe_78_Si_13_B_9_ ribbons could be measured by the optical fiber. We used 502 glue to fix the fiber at both ends of the Fe_78_Si_13_B_9_ ribbons. In this paper, two sensors with two different sensing lengths were fabricated. The sensing lengths were 0.25 m and 1 m, respectively. The sensing lengths were increased by bending the optical fiber back and forth on the ribbons. Multilayer Fe_78_Si_13_B_9_ ribbons formed a whole by overlapping up and down. In this structural design, it was very important to ensure the consistency of the stress state of each layer of the Fe_78_Si_13_B_9_ ribbons. As such, the maximum number of layers of Fe_78_Si_13_B_9_ ribbons used in this paper was two. The sensor structure design will need to be further optimized. 

Based on the thin ribbon characteristics of the Fe_78_Si_13_B_9_, the stiffness could be improved by overlapping materials. Therefore, the multilayer stiffness coupling model between the Iron-based amorphous nanocrystalline ribbons and fiber was established. The elastic modulus was expressed as [26]:

(2) $$E=\left(F/A\right)/\left(\Delta L/L\right),$$

where *E* is the elastic modulus, *F* is the force, *L* is the original length, and Δ*L* is the changed length. Additionally, the tensile stiffness can be expressed as *EA*. To accurately measure the strain of magnetostrictive material caused by magnetic field, the equivalent stiffness relationship should be satisfied: the tensile stiffness of the Fe_78_Si_13_B_9_ should be greater than that of the optical fiber (see Appendix B). 

**Figure 3:**
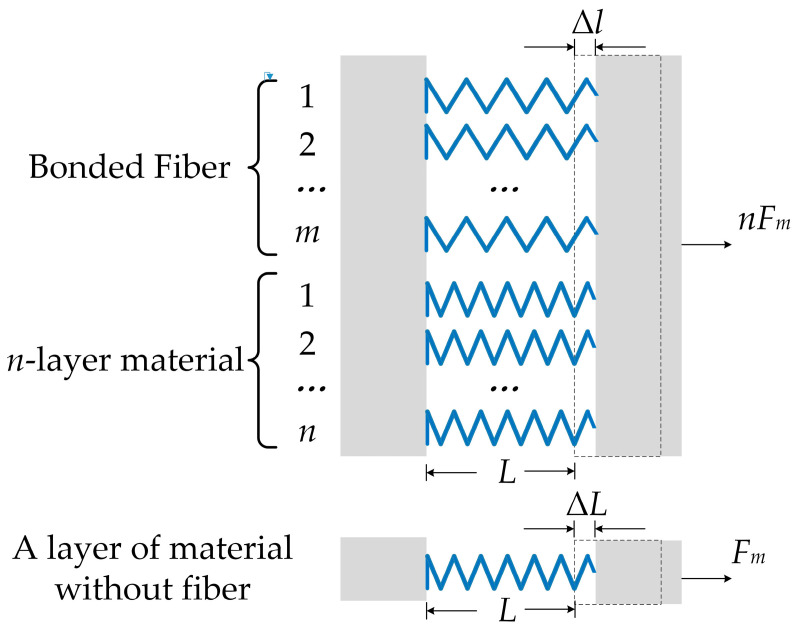
The equivalent stiffness coefficient of the fiber magnetic sensing system.

The optical fiber was pasted back and forth *m* times on the *n*-layer Fe_78_Si_13_B_9_ ribbons, as shown in Figure 3. Therefore, we were able to determine the sensing length as *m* × *L*. Ideally, the strain of the sensing fiber would have been equal to the strain of the Fe_78_Si_13_B_9_ ribbons. However, the Fe_78_Si_13_B_9_ ribbons and optical fiber had different tensile stiffness values. For ease of analysis, the Fe_78_Si_13_B_9_ ribbons and optical fiber were regarded as perfect elastomers, conforming to Hooke’s law, and were represented by simple drawings in Figure 3. For the single-layer Fe_78_Si_13_B_9_ ribbon, the relationship between strain and force under a certain magnetic field was expressed as:

(3) $$F_{m}=\frac{\Delta L}{L}EA,$$

where *F_m_* is the force of single layer Fe_78_Si_13_B_9_ ribbon under certain magnetic fields, *E* is the elastic modulus of the Fe_78_Si_13_B_9_ ribbons, *L* is the length of single layer Fe_78_Si_13_B_9_ ribbon under spring tension, Δ*L* is the length change of single layer Fe_78_Si_13_B_9_ ribbon by magnetostrictive effect, and *A* is the cross section of the Fe_78_Si_13_B_9_ ribbons. The *n*-layer Fe_78_Si_13_B_9_ ribbons and the fiber that was pasted back and forth *m* times pasted on the Fe_78_Si_13_B_9_ ribbons were regarded as a whole, and the relationship between strain and force under a certain magnetic field was expressed as:

(4) $$nF_{m}=\frac{\Delta l}{L}\left(nEA+mE_{f}A_{f}\right),$$

where *E_f_* is the elastic modulus of the optical fiber, *A_f_* is the cross section of the optical fiber, and Δ*l* is the length change of *n*-layer Fe_78_Si_13_B_9_ ribbons and fiber by magnetostrictive effect. We used a spring to pre-stretch the Fe_78_Si_13_B_9_ ribbons to keep them in a tight state. Additionally, the optical fiber was attached to the ribbons under pre-stress. Therefore, all the parameters in the above Equations (3) and (4) are used to describe the magnetostrictive material and optical fiber in the tensile state. 

In an ideal case, the two beams entering the two arms of balance fiber Mach–Zehnder interferometer would be equal. If a magnetic field was then applied, the light beam passing though the sensing arm would be modulated by the magnetostrictive ribbons; its phase modulation Δ*φ* was expressed as [27]:

(5) $$\Delta \varphi =\frac{2\pi n_{eff}\Delta l_{s}}{\lambda_{0}},$$

where *λ*_0_ is the wavelength of light in vacuum, *n*_eff_ is the refractive index of the fiber core, and Δ*l_s_* is the total length change of fiber sensing length. Δ*l_s_* was *m* × Δ*l* in Figure 3. Furthermore, when an external magnetic field was applied to the Fe_78_Si_13_B_9_ ribbons, the Fe_78_Si_13_B_9_ ribbons exhibited axial strain. When Fe_78_Si_13_B_9_ ribbons was just in magnetic saturation, the relationship between the strain and the magnetic field was expressed as [28,29]:

(6) $$\varepsilon_{\max}=\frac{\Delta L_{\max}}{L}=CB^{2},$$

where *C* is the magnetostrictive coefficient of the Fe_78_Si_13_B_9_ ribbons, *B* is the magnetic induction, *ε_max_* is the strain of the Fe_78_Si_13_B_9_ ribbons in the magnetic saturation; *L* is the original length of the Fe_78_Si_13_B_9_ ribbons, and Δ*L_max_* is the length variation in the Fe_78_Si_13_B_9_ ribbons under significant magnetic field conditions. The magnetostrictive strain of the material was not linearly proportional to the magnetic field, which can be roughly divided into three parts [30]:

1. The coefficient *q* increases with the increase in the magnetic field, but the strain increase is small;
2. The coefficient *q* is a fixed value that does not change with the magnetic field, and the strain and the magnetic field are linear;
3. The coefficient *q* decreases with the increase in the magnetic field, and the magnetostrictive material finally reaches saturation.

A certain bias magnetic field can make the magnetostrictive materials work in the linear region, and the relationship between *ε* and *B* was expressed as:

(7) $$\varepsilon =\frac{\Delta L}{L}\approx qB\cos\theta ,$$

where *q* is the coefficient of the Fe_78_Si_13_B_9_ ribbons, which is the strain of magnetostrictive material under unit magnetic field under a bias magnetic field, *ε* is the strain of the Fe_78_Si_13_B_9_ ribbons, *L* is the original length of the Fe_78_Si_13_B_9_ ribbons under a bias magnetic field, Δ*L* is the length variation in the Fe_78_Si_13_B_9_ ribbons, and *θ* is the included angle between the magnetic field and the sensors’ sensitive direction (the sensitive direction of the sensors is the axial direction of the Fe_78_Si_13_B_9_ ribbons, as well as the sensitive direction of the Fe_78_Si_13_B_9_ ribbons and the direction of the optical fiber).

According to Equations (3)–(5) and (7), the relationship between phase and magnetic field was expressed as:

(8) $$\begin{array}{ll} \Delta \varphi & =\frac{2\pi n_{eff}\Delta l_{s}}{\lambda_{0}}\\ & =\frac{2\pi n_{eff}}{\lambda_{0}}m\cdot \Delta l\\ & =\frac{2\pi n_{eff}L}{\lambda_{0}}\cdot \frac{mnEA}{nEA+mE_{f}A_{f}}\cdot qB\cos\theta \end{array}$$

The sensitivity of the fiber magnetic system was expressed as:

(9) $$K=\frac{d\Delta \varphi }{dB}=\frac{2\pi n_{eff}L}{\lambda_{0}}\cdot \frac{mnEA}{nEA+mE_{f}A_{f}}\cdot q\cos\theta ,$$

According to Equation (9), the sensitivity showed a linear correlation between the magnetic field and the phase. We were able to increase the sensing length *m* × *L*, the coefficient *q*, or the layers *n* of the Fe_78_Si_13_B_9_ ribbons to improve sensitivity. 

The parameters of the sensors are shown in Table 1; they were similar to those of giant magnetostrictive materials. The laser source was an NKT narrow linewidth laser with a central wavelength of 1550 nm. The elastic modulus and cross-sectional area of magnetostrictive material and fiber were obtained by tensile test (see Appendix B). The coefficient *q* of the Fe_78_Si_13_B_9_ ribbons was 0.0004 με/μT, which was indirectly estimated during the sensitivity testing of the sensors. The theoretical sensitivities of the fiber optic magnetic field sensors were 0.000574 rad/μT and 0.00222 rad/μT, respectively, which showed that the sensitivity of the optical fiber magnetic field sensor was approximately proportional to the sensing length. In this paper, two sensors with sensing lengths of 0.25 m and 1 m were used to verify the theoretical analysis results. In the future, the sensor will be optimized on this basis, and tens of meters of sensing length will be used to achieve pT-level magnetic field resolution. 

### 2.3. The Principle of Arc Tangent Method

The three channel photodetector signals of the 3 × 3 coupler were as follows:

(10) $$\left\{\begin{array}{c} V_{1}=D_{1}+A_{1}\cos\left[\varphi (t)\right]\\ V_{2}=D_{2}+A_{2}\cos\left[\varphi (t)+\varphi_{1,2}\right]\\ V_{3}=D_{3}+A_{3}\cos\left[\varphi (t)+\varphi_{1,3}\right] \end{array}\right.,$$

where *D*_1_, *D*_2_, and *D*_3_ are the DC components, *A*_1_, *A*_2_, and *A*_3_ are the AC components, *φ*(*t*) is the signal to be detected, and *φ*_1,2_ and *φ*_1,3_ are the phase difference. The signal to be demodulated was expressed as [32]:

(11) $$\varphi \left(t\right)=\arctan\left[\frac{\sqrt{3}\left(V_{2}-V_{3}\right)}{V_{2}+V_{3}-2V_{1}}\right],$$

where we assumed that the three signals were symmetrical, and the phase difference was 120°. In fact, due to the influence of polarization and optical path loss, the assumption was not tenable, which affected the harmonic distortion. 

### 2.4. Experiment Setup

**Figure 4:**
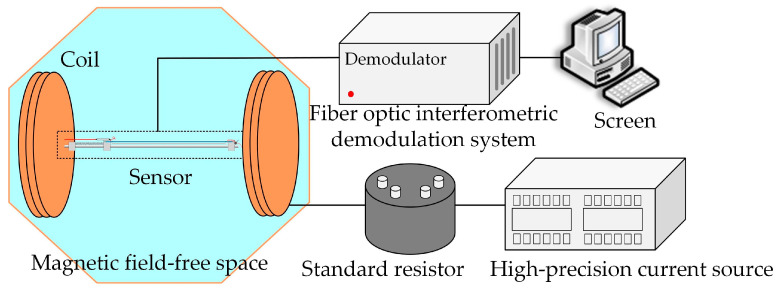
The experimental test diagram of optical fiber magnetic field sensors.

The experimental test diagram is shown in Figure 4. The ring coil in the magnetic field-free space could generate a magnetic field of 0~100 μT through a current source. The standard resistor was connected to the current source to play a monitoring role and to ensure that the input signal was consistent with the set value. The standard resistor was immersed in dry oil, allowing it to better maintain the stability of the resistance state. 

**Figure 5:**
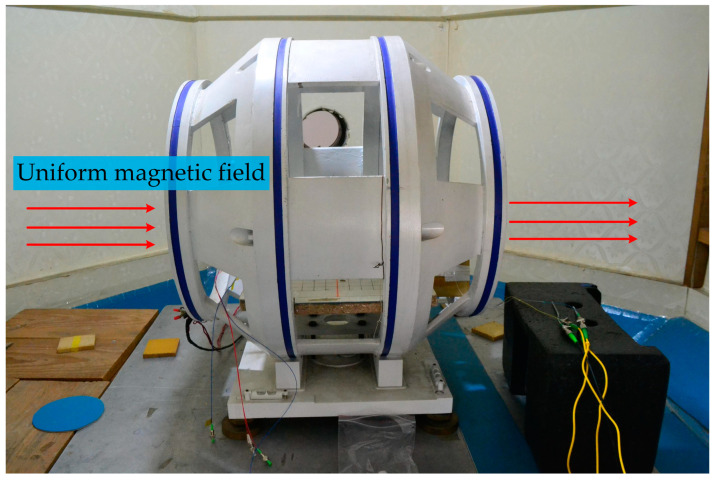
The uniform magnetic field is generated by applying current to the coil in zero magnetic space.

As shown in Figure 5, the coil in the zero magnetic space could generate a uniform magnetic field along the axial direction. The strength and direction of the magnetic field were controlled by the current source, and the current input value was monitored by the standard resistance. 

**Figure 6:**
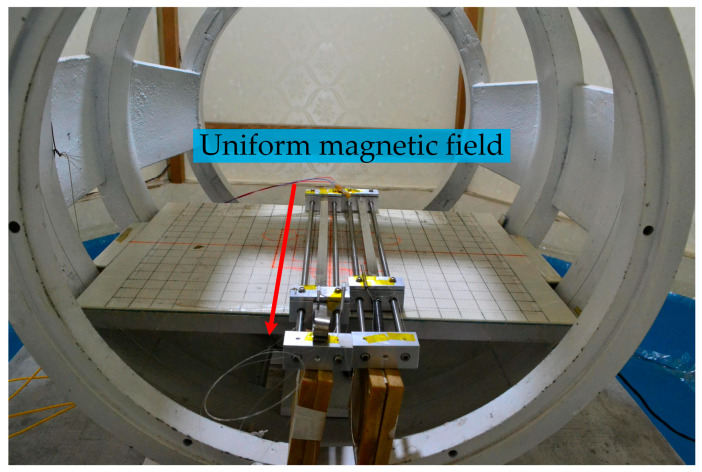
Simultaneous measurement of two optical fiber magnetic field sensors.

As shown in Figure 6, two optical fiber magnetic field sensors were placed in the uniform area of the central magnetic field of the ring coil, and the linearity, frequency response, and directivity of the two sensors were tested. The signals were demodulated and stored using an optical fiber interferometric phase demodulation system. 

## 3. Results and Discussion

The high-precision constant current source was adjusted to make the ring coil produce a bias magnetic field of 100 μT. The original sampling rate of the demodulation system was 20 kHz and the downsampling factor was 4. 

**Figure 7:**
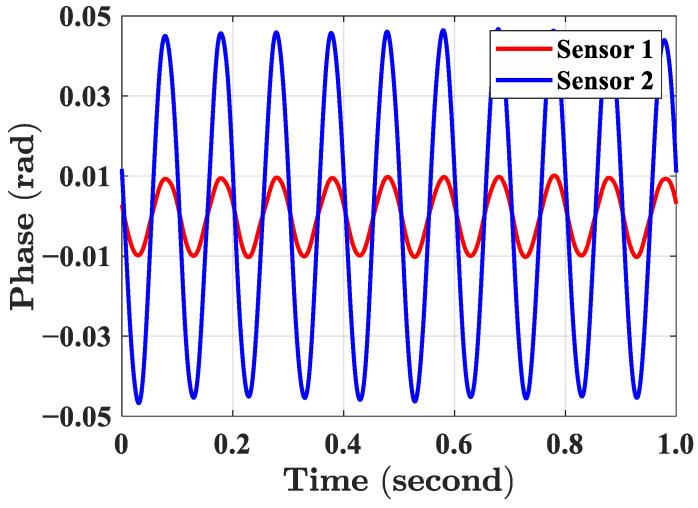
The sinusoidal magnetic field signals collected by two optical fiber magnetic field sensors.

First, we tested that two optical fiber magnetic field sensors were able to respond to the magnetic field and correctly record the magnetic field signal. The current and frequency of the alternating current source were adjusted to generate a sinusoidal AC magnetic field with a frequency of 10 Hz. Figure 7 shows the phase results recorded by two optical fiber magnetic field sensors. The phase showed an obvious sinusoidal waveform, indicating that the sensor responded to the magnetic field generated by the ring coil.

### 3.1. Linearity

**Figure 8:**
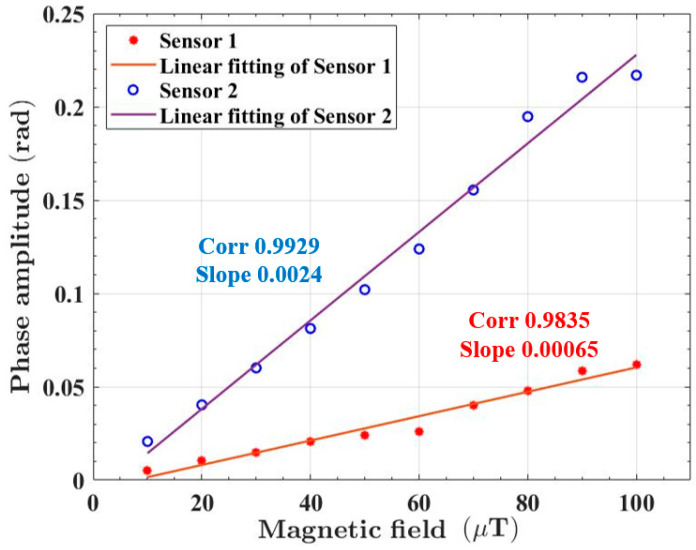
The phase response of optical fiber magnetic field sensors to different magnetic field signals.

The linearity test results are shown in Figure 8. The current and frequency of the alternating current source were adjusted to generate a sinusoidal AC magnetic field with a frequency of 10 Hz. The amplitude of the magnetic field was increased from 0 μT to 100 μT with a step of 10 μT. 

The linear fitting of the magnetic field response results of the first sensor showed that the correlation coefficient was 0.9835 and the slope was 0.00065, indicating that the sensitivity of the first sensor was about 0.00065 rad/μT. The linear fitting of the magnetic field response results of the second sensor showed that the correlation coefficient was 0.9929 and the slope was 0.0024, indicating that the sensitivity of the second sensor was about 0.0024 rad/μT. The sensitivities of the sensors were consistent with expectations. However, the Fe_78_Si_13_B_9_ ribbons could have a higher magnetic field response at a lower frequency (below 10 Hz). In addition, the bias magnetic field was set to 100 μT in this test, but the optimal bias magnetic field of the Fe_78_Si_13_B_9_ ribbons was not determined. 

### 3.2. Frequency Response

The current and frequency of the alternating current source were adjusted to generate a sinusoidal AC magnetic field with an amplitude of 100 μT. The magnetic field frequencies were selected as 10 Hz, 15 Hz, 20 Hz, 30 Hz, 50 Hz, 80 Hz, 100 Hz, 120 Hz, 150 Hz, and 200 Hz, respectively. 

**Figure 9:**
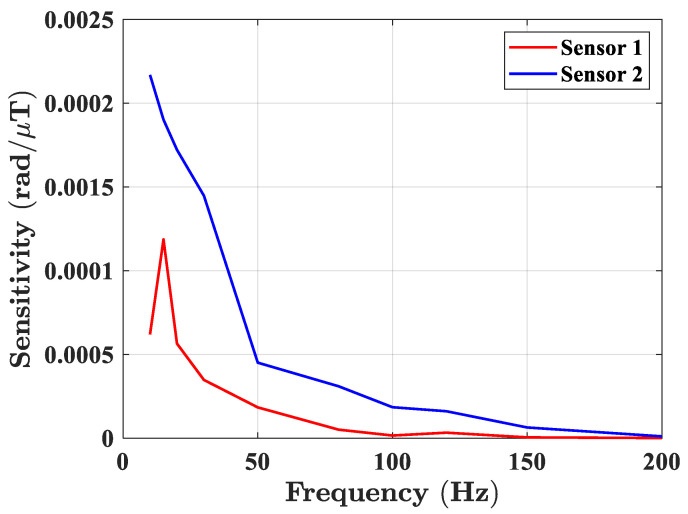
The frequency response of optical fiber magnetic field sensors.

The frequency response test results are shown in Figure 9. The first optical fiber magnetic field sensor had a sensitivity peak near 15 Hz, and then the response grew smaller as the frequency increased. The phase response of the second optical fiber magnetic field sensor decreased monotonously with the increase of frequency. The resonant frequency of the first sensor was around 15 Hz, while the resonant frequency of the second sensor could have been less than 10 Hz due to the different fiber sensing length. Herein, two optical fiber magnetic field sensors with different sensing lengths were used. One of these used a layer of ribbon, and the fiber was pasted on a ribbon length. Another sensor used two layers of the Fe_78_Si_13_B_9_ ribbons, and the fiber was pasted four ribbon lengths back and forth. Therefore, the equivalent stiffnesses of the two sensors were different, as were the mechanical resonance frequencies. The frequency response of the sensor was greatly affected by its structure. Therefore, the peak in the frequency response curve should have been the mechanical resonance frequency of the sensor, while the mechanical resonance frequency of the other sensor was not in the frequency range. 

### 3.3. Direction

**Figure 10:**
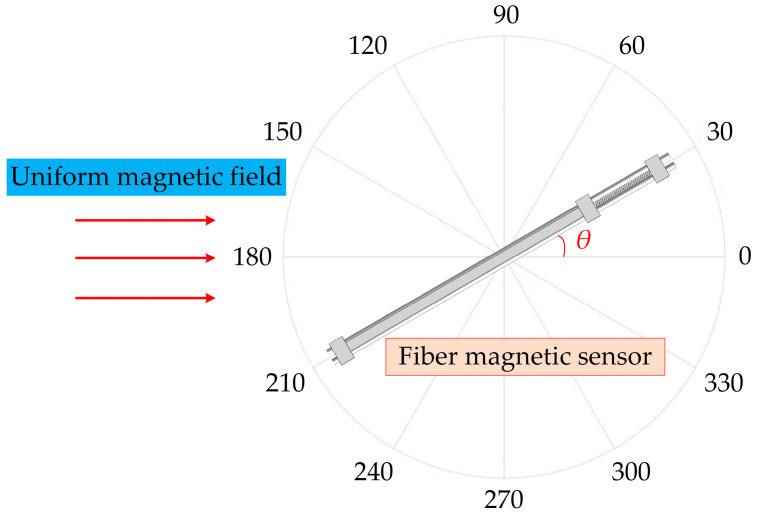
Directivity test of optical fiber magnetic field sensors.

When the magnetic field sensitive direction of the Fe_78_Si_13_B_9_ ribbons was consistent with the axial direction of the ring coil, the magnetostrictive effect was the most obvious and the largest response of the optical fiber magnetic field sensor was observed. As shown in Figure 10, when there was an angle *θ* between the magnetic field sensitive direction of the Fe_78_Si_13_B_9_ ribbons and the axial direction of the ring coil, the phase response could be expressed as:

(12) $$\Delta \varphi_{\theta }=\Delta \varphi *\cos\theta ,$$

where ∆*φ* is the phase response of the sensor when the angle is 0°, and Δ*φ_θ_* is the phase response of the sensor when the angle is *θ*. 

**Figure 11:**
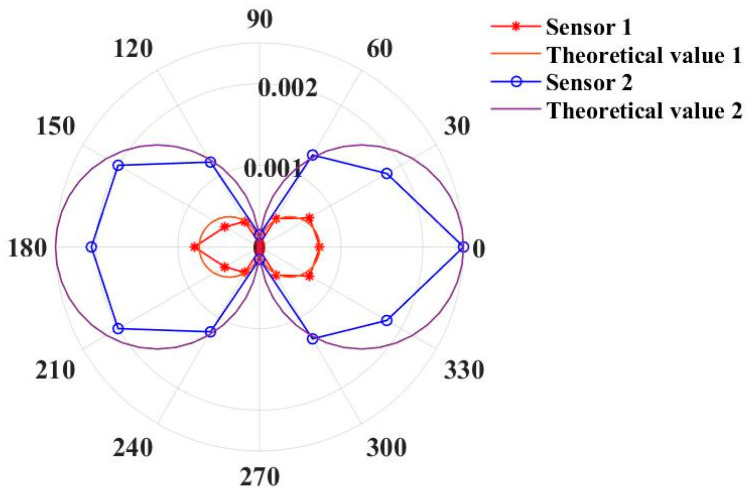
The directivity test results of optical fiber magnetic field sensors.

The current value of the alternating current source was adjusted to generate a sinusoidal AC magnetic field with an amplitude of 100 μT. The magnetic field frequency was set to 10 Hz. The angle between the optical fiber magnetic field sensor and the axial direction of the ring coil was adjusted counterclockwise with 30° as the step from 0°. The angle test range was from 0° to 180°. The directivity test results are shown in Figure 11.

The responses of the two sensors to the magnetic field were the weakest at 90°, which was consistent with the cosine change. There was a deviation between the sensitivity measurement results and the theoretical values. The main reason for this was that the airflow and the change in temperature generated by people exerted a great influence when adjusting the angle. 

### 3.4. Phase Resolution Test of Demodulation System

**Figure 12:**
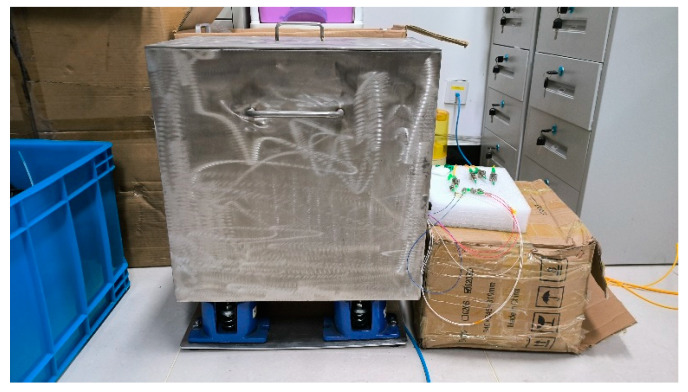
Vibration isolation box is used for phase resolution test.

**Figure 13:**
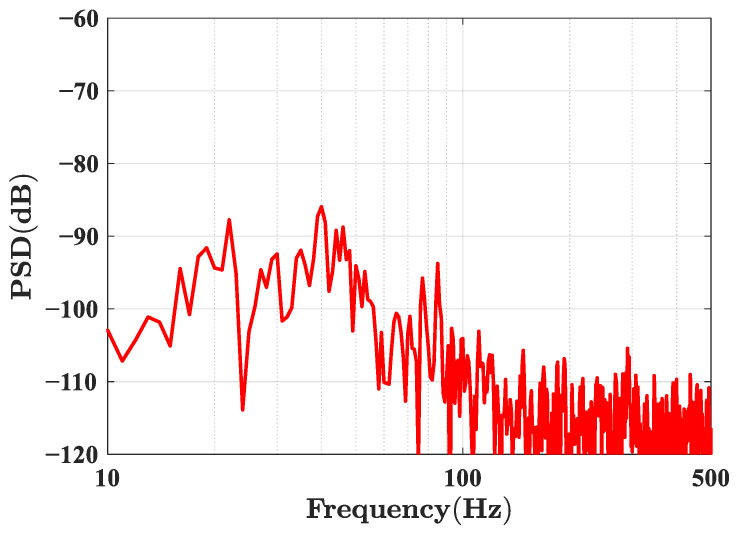
The phase power spectral density estimation.

As shown in Figure 12, a 3 × 3 equal-arm Mach–Zehnder interferometer was placed on the spring vibration isolation plate in the vibration isolation box. Four optical fiber jumpers were drawn from the hole on one side of the vibration isolation box and connected with the demodulation system. After the double-layer covers of the vibration isolation box were closed, the structure of the vibration isolation box remained stable for a significant time. The phase noise power spectral density estimation was calculated according to the 3 × 3 demodulation results. Data for a length of time of 100 s time were selected for power spectral density estimation; the results are shown in Figure 13. 

According to the power spectral density estimation, the phase resolution was less than 1 × 10^−5^ rad/√Hz @ 10 Hz. The environmental noise was not completely suppressed, so there was significant environmental interference near 20 Hz and 40 Hz. According to Equation (1), the magnetic field resolutions of the two sensors, respectively, were 15.4 nT/√Hz @ 10 Hz and 4.2 nT/√Hz @ 10 Hz. 

Table 2 presents a performance comparison of the optical fiber magnetic field sensors based on the magnetostrictive effect. Active demodulation methods such as lock-in-amplification could achieve high magnetic field resolution, but the methods would also limit the advantages of passive optical fiber sensing systems. For a passive optical fiber magnetic field sensor, we achieved nT-level magnetic field resolution at low frequency. Since the longest sensing length of sensors in this paper was only 1 m, further improvement in the magnetostrictive response of a sensor could be obtained through improvement of the sensor structure and enhancement of the sensing length. 

## 4. Conclusions

In this paper, an optical fiber magnetic field sensor structure was designed based on iron-based amorphous nanocrystalline ribbons. Two optical fiber magnetic field sensors with sensing lengths of 0.25 m and 1 m, respectively, were calibrated. The linearity, frequency response, and directivity of the two sensors were tested. The sensor was suitable for low frequency measurement, and the sensitivity decreased as frequency increased. The directivity of the sensor conformed to the cosine function. Under a bias magnetic field of 100 μT, the sensitivities of the two sensors were 0.00065 rad/μT and 0.0024 rad/μT, respectively, which verified the sensitivity multiplication relationship between the two sensors. Based on the sensitivity theoretical analysis and experimental results, it would be feasible to build an ultra-long sensing length fiber magnetic field sensor to achieve pT level magnetic field resolution. 

## Figures and Tables

**Table 1:** Designed parameters.

Parameter	Sensor 1	Sensor 2
*n_eff_* [31]	1.4682	1.4682
*λ*_0_	1550 nm	1550 nm
*m* × *L*	1 × 0.25 m	4 × 0.25 m
*Q*	0.0004 με/μT	0.0004 με/μT
*N*	1	2
*E*	85.75 GPa	85.75 GPa
*E_f_*	83.8 GPa	83.8 GPa
*A*	0.3325 mm^2^	0.3325 mm^2^
*A_f_* [27]	0.0123 mm^2^	0.0123 mm^2^
*K* (*θ* = 0)	0.000574 rad/μT	0.00222 rad/μT

**Table 2:** Performance comparison of optical fiber magnetic field sensors based on the magnetostrictive effect.

References	Methods	Sensitivity	System Resolution	Magnetic Field Resolution
[21]	Mach–Zehnder interferometer Coated Fiber Lock-in-amplifier	0.0091 rad/μT	5.6 × 10^−4^ rad/√Hz @ 245 Hz	62 nT/√Hz @ 245 Hz
[24]	Michelson interferometer Lock-in-amplifier	0.004 rad/μT	6 × 10^−7^ rad/√Hz @ 0.1 Hz	150 pT/√Hz @ 0.1 Hz
[25]	Michelson interferometer Lock-in-amplifier	105.9 μV/nT	1.03 × 10^−5^ V/√Hz @ 1 Hz	0.1 nT/√Hz @ 1 Hz
[22]	Mach–Zehnder interferometer Passive demodulation	0.06983 rad/μT	9.17 × 10^−5^ rad/√Hz @ 200 Hz	2.14 nT/√Hz @ 200 Hz
This paper	Mach–Zehnder interferometer Passive demodulation	0.00065 rad/μT 0.0024 rad/μT	1 × 10^−5^ rad/√Hz @ 10 Hz	15.4 nT/√Hz @ 10 Hz 4.2 nT/√Hz @ 10 Hz

**Figure A1:**
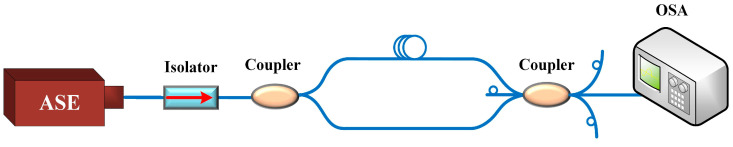
Optical fiber interferometer arm length difference measurement.

**Figure A2:**
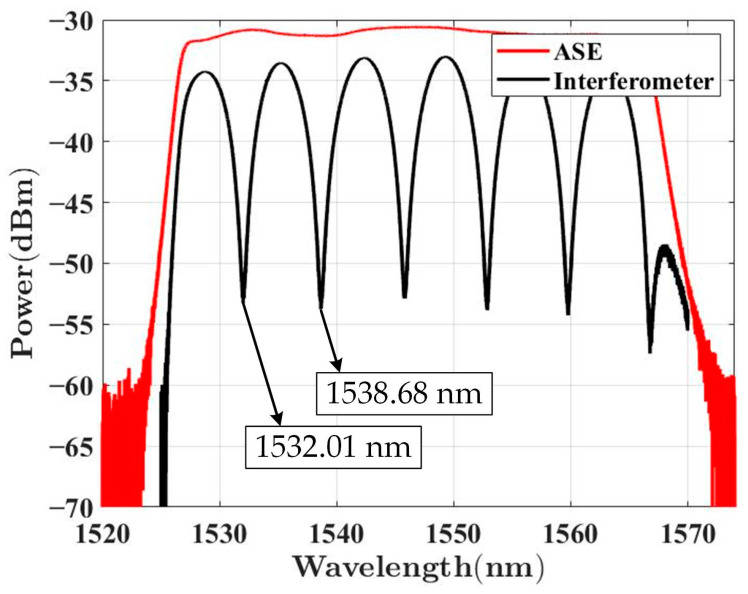
ASE light source spectrum and interferometer spectrum.

**Figure A3:**
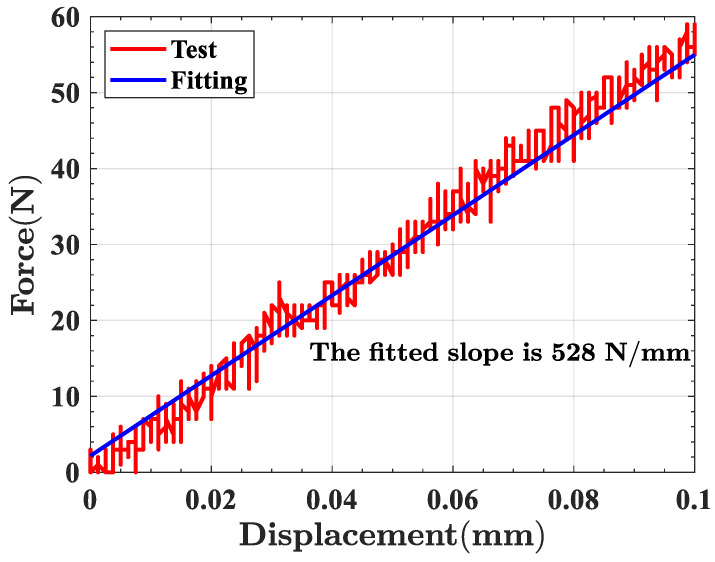
The displacement and tensile test results of the Fe_78_Si_13_B_9_ ribbons.

**Figure A4:**
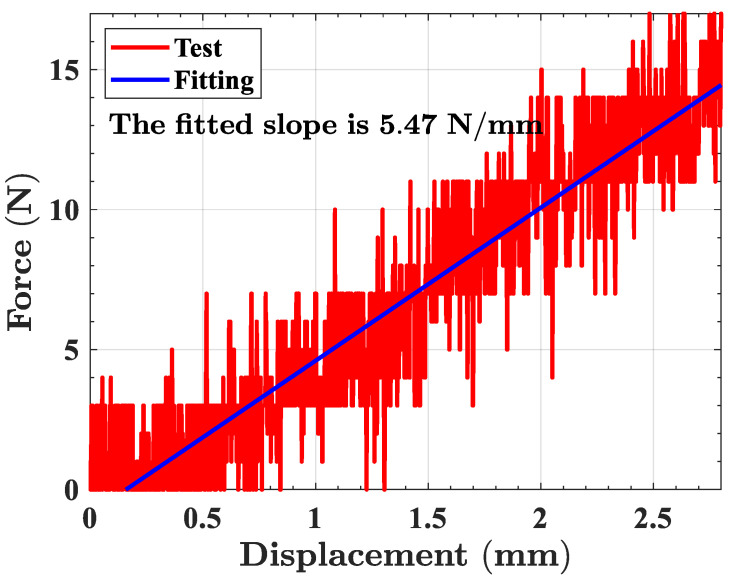
The displacement and tensile test results of the optical fibers.

## Data Availability

The data are not publicly available due to the Confidentiality and Non-disclosure Agreement with the funders.

## Appendix A

The interference spectrum of the interferometer was measured by a spectrometer, and the arm length difference was calculated by the wavelength interval at the adjacent valley power. The measurement system is shown in Figure A1.

The relationship between wavelength interval and the wavelength corresponding to the minimum value can be expressed as [33]:

(A1) $$\Delta \lambda =\frac{\lambda_{1}\lambda_{2}}{n\Delta L},$$

where Δ*λ* is the wavelength interval, *λ*_1_ and *λ*_2_ are the wavelength corresponding to the minimum value, *n* is the refractive index of optical fiber, and Δ*L* is the arm length difference of the interferometer.

The spectrum of a sensor was shown in Figure A2, and the interferometer arm length difference can be obtained according to Equation (A1). *λ*_1_ was 1532.01 nm and *λ*_2_ was 1538.68 nm in Figure A2. Then, we could find that Δ*L* was 0.241 mm. In the same way, the arm length difference of another sensor was measured and was close to 1 mm. Therefore, the interferometers of both sensors can be regarded as equal-arm interferometers.

## Appendix B

The displacement and tensile test of magnetostrictive material and optical fiber were carried out by electronic pressure testing machine, and the elastic modulus was calculated. According to Equation (2), the relationship between force and deformation can be expressed as:

(A2) $$k=\frac{F}{\Delta L}=\frac{EA}{L},$$

where *k* is the fitting slope of force and deformation. The tensile test results of the Fe_78_Si_13_B_9_ ribbons and the optical fibers are shown in Figure A3 and Figure A4.

The length of the tested Fe_78_Si_13_B_9_ ribbons was 54 mm, the width was 9.5 mm, and the thickness was 0.035 mm. Moreover, according to Equation (2), the elastic modulus and tensile stiffness of the Fe_78_Si_13_B_9_ ribbons can be calculated as 85.75 GPa and 28512 N.

The length of the tested optical fiber was 188 mm and the diameter was 125 μm [27]. Additionally, the elastic modulus and tensile stiffness of the optical fiber can be calculated as 83.8 GPa and 1028 N. The tensile stiffness of the Fe_78_Si_13_B_9_ ribbons is greater than that of optical fiber. The strain of the Fe_78_Si_13_B_9_ ribbons caused by magnetic field can be measured by optical fiber.
